# 936 Frostbite and Social Determinants of Health: A Scoping Review

**DOI:** 10.1093/jbcr/iraf019.467

**Published:** 2025-04-01

**Authors:** Gursagar Jhanji, Abby Rentz, Justin Gawaziuk, Sarvesh Logsetty, Rae Spiwak, Brenda Comaskey

**Affiliations:** University of Manitoba; Manitoba Firefighters Burn Unit; University of Manitoba; Manitoba Firefighters Burn Unit; University of Manitoba; University of Manitoba

## Abstract

**Introduction:**

Frostbite is a type of cold thermal injury caused by several mechanisms, including direct cellular injury and indirect injury from ischemia and reperfusion. While there has been considerable emphasis on the physiology and treatment of frostbite, there has been a lack of extensive research investigating the impact of Social Determinants of Health (SDoH) on frostbite injuries. SDoH are non-medical factors that impact health, such as income, housing, and childhood environment. These factors can influence health inequities and have been shown to impact health more than healthcare or lifestyle. Addressing this gap would provide us valuable insights for developing effective intervention and prevention initiatives.

**Methods:**

We conducted a scoping review guided by the methodology framework of Arksey and O’Malley and the Preferred Reporting Items for Systematic Reviews and Meta-analysis Protocols Extension for Scoping Reviews Guidelines (PRISMA-ScR). The MedLine database was searched to identify studies that met our review criteria. Studies of various designs and methodologies published since January 1, 2000 that examine SDoH in relation to frostbite injury in adults aged 18 or older were considered for inclusion in this review.

**Results:**

The search identified 484 studies, 24 of which were retained in the final review. The majority of the manuscripts identified a SDoH with substance use disorder (n=15), older age (n=10), living with a mental disorder (n=12), experiencing homelessness (n=9) and male sex (n=19) being key determinants in the context of frostbite.

**Conclusions:**

In conclusion, we have illustrated how social determinants of health such as substance use disorder, experiencing homelessness older age, male sex, and a psychiatric history can impact one’s risk of acquiring a frostbite injury. Furthermore, these factors should not be seen in isolation of each other but understood in the context of a Venn diagram, with each factor as a circle and with each overlap of the circles increasing the individual’s risk of harm exponentially.

**Applicability of Research to Practice:**

Understanding predictors of frostbite injury provides the potential to facilitate early interventions and treatments, which may decrease severity of injury and guide public health programs. Interventions informed by this study can potentially modify the trajectory of future health and social outcomes in individuals.

**Funding for the Study:**

Foundation funding

